# Evaporative Cooler Use Influences Temporal Indoor Relative Humidity but Not Dust Mite Allergen Levels in Homes in a Semi-Arid Climate

**DOI:** 10.1371/journal.pone.0147105

**Published:** 2016-01-25

**Authors:** James D. Johnston, Steven C. Tuttle, Morgan C. Nelson, Rebecca K. Bradshaw, Taylor G. Hoybjerg, Julene B. Johnson, Bryce A. Kruman, Taylor S. Orton, Ryan B. Cook, Dennis L. Eggett, K. Scott Weber

**Affiliations:** 1 Department of Health Science, Brigham Young University, Provo, Utah, United States of America; 2 Department of Microbiology & Molecular Biology, Brigham Young University, Provo, Utah, United States of America; 3 Department of Statistics, Brigham Young University, Provo, Utah, United States of America; Ecole des Mines d'Alès, FRANCE

## Abstract

Concerns about energy consumption and climate change make residential evaporative coolers a popular alternative to central air conditioning in arid and semi-arid climates. However, evaporative coolers have been shown to significantly increase indoor relative humidity and dust mite allergen levels in some studies, while showing no association in other studies. Improved measurement of temporal fluctuations in indoor relative humidity may help identify factors that promote mite growth in homes in dry climates. Dust samples and continuous indoor relative humidity measurements were collected from homes with central air conditioning and homes with evaporative coolers in Utah. Samples were collected over two seasons, winter/spring (Jan–Apr) and summer (July–Sept), 2014. Dust samples were analyzed for Der p 1 and Der f 1 using a two-site monoclonal antibody-based enzyme-linked immunosorbent assay (ELISA) analysis. Housing characteristics including age of home, occupant density, and age of mattresses, furniture, and carpeting were also measured. Positive Der p 1 or Der f 1 samples were found in 25.0% of the homes and there was no difference in mean allergen levels by type of air conditioning. Indoor relative humidity was significantly higher in homes with evaporative coolers compared to those with central air conditioning during the summer. Homes with evaporative coolers also spent significantly more time during summer above 55.0% and 65.0% relative humidity compared to central air homes, but not above 75.0%. Findings from this study suggest that increased humidity from evaporative coolers may not be sufficient to exceed the critical equilibrium humidity or maintain humidity excursions for sufficient duration in relatively larger single-family homes in semi-arid climates to support mite growth and reproduction.

## Introduction

Exposure to house dust mite (HDM) allergens is associated with immune system sensitization; development of asthma, rhinitis, and atopic dermatitis; and exacerbation of disease symptoms in sensitized individuals [1–7]. D*ermatophagoides farinae* and *D*. *pteronyssinus* are the most prevalent mite species found in homes in humid temperate regions in the U.S. and worldwide [8, 9]. These mite species acquire water primarily by absorbing moisture from ambient air; thus, indoor relative humidity (RH) is a critical determinant of mite population growth [10–12]. Due to low RH in arid and semi-arid regions, HDMs are usually absent in homes unless indoor humidity is artificially raised [8, 13, 14]. The minimum RH required to prevent mites from desiccating, known as the critical equilibrium humidity, ranges from 55.0–75.0% RH at 15.0–35.0°C [10, 15]. Thus, maintaining indoor RH below 50.0% year-round is recommended as a primary control strategy to avoid HDM growth and subsequent allergen exposures [2].

Evaporative cooling is a common form of energy-efficient, low-cost air conditioning used during summer months in arid and semi-arid regions [16]. Evaporative “swamp” coolers work by pulling hot, dry outdoor air into the home across a wet medium, usually natural or synthetic fiber pads. As water in the fiber pads evaporates it draws energy, in the form of sensible heat, from the outdoor air. In this adiabatic process, indoor air temperature is reduced as sensible heat from outdoor air is transferred to latent heat in the form of water vapor [17]. Consequently, homes with evaporative coolers can experience significantly higher RH during summer months. For instance, Ellingson et al. (1995) found that RH in Colorado homes with evaporative coolers was 16.0% higher on average in the summer compared to homes with no air conditioning in the same geographical area (p < 0.001) [18].

Moisture introduced into the home by evaporative coolers may create conditions that support HDM growth in regions where they are normally absent. Nelson and Fernandez-Caldas (1995) found low numbers of mites in homes in the Rocky Mountain states where ambient RH is generally low; however, a high number of *D*. *farinae* (3,000 mites/g of house dust) was found in one of the four homes where evaporative coolers were used [13]. Similarly, studies in both southeastern Australia and Colorado, USA found that homes with evaporative coolers were significantly more likely to test positive for HDM allergens Der p 1 and Der f 1 [18, 19]. Furthermore, Prasad et al. (2009) found that children living in homes with evaporative coolers in Nevada, USA were significantly more likely to show skin reactivity to mite allergens compared to those living in homes with central or no air conditioning [20].

The relationship between evaporative cooler use and HDM allergen levels is not well understood. This may be partially due to limitations in RH measurement strategies used in previous studies. Vanlaar et al. (2001) reported humidity data from outdoor centralized monitoring stations, but indoor RH was not measured [19]. Nelson and Fernandez-Caldas (1995) did not report indoor RH. Ellingson et al. (1995) estimated mean indoor RH using a participant-collect strategy, where daily single-point measures were collected during evening hours [18]. Single-point measures, however, do not capture temporal RH variation within the home, and are poorly correlated with mean indoor RH over multiple days [21]. Additionally, mean indoor RH may not be the most appropriate measure for evaluating in situ critical equilibrium conditions for mites in dry climates. For example, under laboratory conditions of 36.0% RH and 16.0°C, female *D*. *pteronyssinus* mites can survive and produce eggs given moist air excursions of 76% RH for as little as 1.5–3.0 hrs/day [22]. Continuous monitoring over multiple days has the advantage of showing mean RH as well as short-term moisture excursions that may contribute to mite growth, but to date this sampling strategy has not been used in studies of HDMs in homes with evaporative coolers.

Concerns about energy consumption, sustainability, and climate change make evaporative cooling a popular alternative to central air conditioning in arid and semi-arid climates, such as the southwest U.S. and Rocky Mountain states [23, 24]. However, the energy-saving advantages of evaporative cooling should be considered in the context of human exposure to mite allergens, particularly among young children. Understanding the relationship between evaporative cooler use and indoor RH trends may help guide future efforts to limit mite growth and subsequent allergen exposures in homes with evaporative coolers.

In this study, we evaluated levels of Der p 1 and Der f 1 in single family homes in Utah County, Utah, which has a semi-arid climate with hot, dry summers and cold winters. The climate in Utah County provides a model environment to test the role of central air conditioning and evaporative coolers on indoor RH and dust mite allergen levels since the outdoor RH is not naturally within the critical equilibrium humidity range for mite growth. Based on previous studies in Colorado [18] and Nevada [20] with similar climates, we hypothesized that homes with evaporative coolers would have significantly higher levels of Der p 1 and Der f 1 than homes with central air conditioning. We found that mean indoor RH was significantly higher in homes using evaporative coolers during summer months, but levels were below the critical equilibrium humidity necessary for optimal dust mite growth. Using a continuous monitoring strategy, we also evaluated the number of minutes per day homes were at or above pre-determined RH levels. Homes with evaporative coolers spent significantly more time above 55.0% and 65.0% RH per day compared to homes with central air conditioning; however, there was no significant difference in the amount of time spent above 75.0% RH. Our measurements of Der p 1 and Der f 1 found low levels of allergen in both the air-conditioned and evaporative cooler homes. Allergen levels appeared to be most strongly associated with age of home.

## Materials and Methods

### Study Population

Study homes (N = 46) were recruited from among faculty and staff at Brigham Young University in Provo, Utah USA by means of a flyer distributed through campus mail. Participant homes were at least 5 years old (build prior to 2009) and located in Utah County, Utah (approx. elev. 4560 ft (1390 m)). Homes in which humidifiers and vaporizers were used were excluded from the study. Homes with prior water damage, defined as areas greater than 10 ft^2^ (0.93 m^2^), were also excluded from the study. Quota sampling was used until 23 homes with central air conditioning and 23 homes with evaporative coolers were recruited into the study. Reservoir dust samples and indoor temperature and RH measurements were collected first during winter/spring (Jan–Apr) and again in summer (Aug–Sept), 2014. Winter/spring dust samples were collected to determine baseline mite allergen levels prior to summer air conditioner use. During the winter/spring visit study personnel obtained written consent and administered a questionnaire on home characteristics. Brigham Young University’s Institutional Review Board (IRB) approved this study (IRB #X130327).

### Temperature and Humidity Measurement

Indoor air temperature and RH measurements were collected continuously for approximately 72 hrs in each home during both winter/spring and summer sampling periods. Measurements were collected using five Extech SD500 humidity/temperature dataloggers (Extech Instruments, Corp., Waltham, MA, USA). NIST-traceable calibration of temperature and humidity sensors was performed prior to data collection. All instruments were within manufacturer’s tolerances of ±0.8°C (1.5°F) and ±4%RH. Dataloggers were placed 0.91–1.83 m (3–6 ft) above the floor and away from cooling/heating vents in a central living area in the home, and set to record temperature and RH every 5 min during the sampling period. Outdoor air temperature and relative humidity measurements were collected from a weather monitoring station located on the campus of Brigham Young University.

### Reservoir Dust Collection

Reservoir dust samples were collected from four areas in each home: the homeowner’s mattress, the floor adjacent to the mattress, upholstered furniture in a main living area of the home, and the floor adjacent to the upholstered furniture. Dust samples were collected using a Eureka (Bloomington, IL, USA) “Mighty Mite” vacuum (model 3684F) fitted with a Duststream^®^ dust collector and 40 μm nylon mesh filter (Indoor Biotechnologies, Charlottesville, VA, USA). Samples were collected from each surface by vacuuming within a taped 1m^2^ area for 3 min. We sieved dust samples through a #50 wire mesh (300μm), transferred to 15 ml polypropylene conical tubes, and stored at -20° C until analyzed.

### Sample Analysis

Reservoir dust samples were analyzed for house dust mite allergens Der p 1 and Der f 1. Allergen extraction was performed by suspending 100 mg of dust in 2 mL of phosphate-buffered saline with 0.05% Tween-20 (PBS-T), followed by agitation at room temperature for 2 hrs. Samples were then centrifuged at 2500 RPM at 4°C for 20 min and supernatants were collected and stored at -20°C until enzyme-linked immunosorbent assay (ELISA) analysis. Sample extracts (200 μL) were added in triplicate to each plate. A 10-point curve was made using sequential two-fold dilutions of a known standard in PBS-T with 1% BSA. Allergen levels were measured using two-site monoclonal antibody-based ELISA kits for Der p 1 and Der f 1 (Indoor Biotechnologies, Charlottesville, VA, USA). Quality control dust samples were obtained from a reference laboratory (DACI, Johns Hopkins University, Baltimore, MD, USA) and run on each plate as an internal control of laboratory performance of ELISA analyses. The reference dust contained known levels of Der f 1 (1.03 μg/g), Der p 1 (1.40 μg/g) [25]. Samples were read with an optical density plate reader at wavelength 405 nm. Prism 6 software was used to extrapolate allergen concentrations from the standard curve for each plate. The limit of detection for both Der p 1 and Der f 1 assays were 0.04 μg/g of dust.

### Housing Questionnaire

During the winter/spring sampling visit, study personnel administered a survey to collect information about features of the home. Survey items included age of home, square footage, number of residents, and age of mattress, furniture, and carpet (if applicable). Occupant density, reported as number of people per 1000 square feet, was calculated from the number of residents and square footage of each home. To control for mite allergens that may have been introduced to study homes, the survey also included questions to determine if mattresses or upholstered furniture were moved to Utah County from locations with high ambient RH.

### Statistical Analysis

The distribution of allergen levels was positively skewed. To normalize the distribution we log transformed the values. From the log transformed data we performed mixed models ANCOVA of allergen levels for the housing factors as well as season and type of air conditioning. The results were back transformed using the antilog to report geometric mean allergen levels and 95% confidence intervals. Comparisons of indoor temperature and RH by season and type of air conditioning were evaluated using t–tests with post-hoc Tukey-Kramer analysis. Mean temperature and RH were calculated by first dividing the day into 288 time points per 24-hr period (00:00–23:55). Mean temperature and RH were then calculated for each of these 288 time points to estimate averages for a given time of day across the winter/spring and summer sampling periods. The proportion of time at or above a specific percent RH for each home was calculated by counting the number of observations at or above that level and dividing by the total number of observations for that home. From this proportion we calculated the number of minutes that each home was at or above 55%, 65%, and 75% during a twenty-four hr day. We performed an ANCOVA to evaluate the interaction between season and type of air conditioning as well as housing factors associated with HDM allergen levels. All analyses were performed using SAS, version 9.3 (SAS institute Inc., Cary, NC, USA). An alpha level of 0.05 was used to determine statistical significance.

## Results

This study evaluated levels of Der f 1 and Der p 1 in single family homes in Utah County, Utah, which has a semi-arid climate not conducive to robust dust mite growth. Of the study homes, 22 with central air conditioning and 18 with evaporative coolers completed both winter/spring and summer sampling. Home characteristics are shown in Table 1. On average, homes with evaporative coolers were older (t_38_ = 4.11, p < 0.001) and had fewer square feet (t_36_ = 2.69, p = 0.011) than homes with central air conditioning. Homes did not differ significantly in number of residents or occupant density, or by age of mattresses, furniture, or floor coverings. During the winter/spring sampling period the mean daily outdoor temperature and RH were 4.65°C and 49.9% respectively. During the summer sampling period the mean daily outdoor temperature and RH were 20.89°C and 42.2% respectively. Mean maximum outdoor temperatures on days when homes were sampled during the summer were 30.37°C and 30.52°C for central air and evaporative cooler homes, respectively. Mean maximum outdoor temperature during summer did not differ significantly between when central air and evaporative cooler homes were sampled (t_38_ = 0.19, p = 0.8513).

**Table 1:** Characteristics of single-family homes with central air conditioning and evaporative coolers in Utah County, Utah (N = 40).

Home Characteristics	Central Air Conditioning (n = 22)				Evaporative Cooler (n = 18)				P-value^a^
	Mean	s.d.	Min	Max	Mean	s.d.	Min	Max	
Age of home (yrs)	27.8	24	6	116	62	28.6	30	121	<0.001
Square footage	3047	916	1250	4770	2277	843	930	3600	0.011
Number of residents	4.6	1.9	2	8	4.1	2.9	1	11	0.482
Occupant density^b^	1.6	0.6	0.4	2.9	1.9	1.5	0.6	6.7	0.398
Mattress age	7.8	4.8	2	20	11	8.5	1	31	0.13
Furniture age	10.4	13.5	1	60	12.1	6.3	1.5	25	0.615
Bedroom carpet age^c^	13.4	9.7	3	44	11.5	9.2	0.3	36	0.529
Living room carpet age	10.9	9.1	1.5	44	10	9.8	0.2	40	0.763

^a^ p-values based on t-tests.

^b^ Occupant density calculated as number of people living in the home per 1000 square feet.

^c^ Two homes were excluded from this analysis because the bedroom floors were wood rather than carpet. One excluded home used a central air conditioner and the other used an evaporative cooler.

Both Der p 1 and Der f 1 allergens were detected at low levels during both seasons. Prior to log transforming the data, only one sample from one home had mite allergen levels greater than 2 μg/g dust, the commonly accepted threshold for sensitization [26, 27]. Based on the limit of detection of 0.04 μg/g of dust, 25/292 (8.6%) samples were positive for one or both allergens. The overall geometric means and 95% confidence intervals for positive samples over the course of the study were 0.108 μg/g of dust (0.072, 0.145) for Der p 1 and 0.369 μg/g of dust (0.00, 1.380) for Der f 1, respectively. Allergen levels by location and season are shown in Table 2. Of the positive samples, 19 (76.0%) contained Der p 1 and six (24.0%) contained Der f 1. For Der p 1 there were 14 positive samples from five homes with central air conditioning, and five positive samples from four homes with evaporative cooling. For Der f 1, there were four positive samples from two homes with central air conditioning, and two positive samples from one home with evaporative cooling.

**Table 2:** Reservoir dust levels of Der p 1 and Der f 1 in Utah homes by location and season.

	Winter/Spring (Jan–Apr)				Summer (Aug–Sept)			
	Family Room Floor	Upholstered Furniture	Bedroom Floor	Mattress	Family Room Floor	Upholstered Furniture	Bedroom Floor	Mattress
Der p 1								
No.(+) Samples	3	1	1	5	2	3	1	3
Mean^A^	0.106	0.148	0.298	0.093	0.08	0.043	0.066	0.121
Min./Max	(0.045, 0.144)			(0.041, 0.208)	(0.078, 0.082)	(0.041, 0.048)		(0.067, 0.175)
Der f 1								
No.(+) Samples	0	0	1	3	0	0	0	2
Mean^A^			1.381	0.136				0.046
Min./Max				(0.046, 0.198)				(0.042, 0.050)

^A^ Values are geometric means expressed in μg Der p 1/Der f 1 per gram of sieved dust. Of the 40 homes completing both winter/spring and summer sampling periods, 10 (25.0%) tested positive for mite allergens. For Der p 1, 14 of the positive samples originated in 5 homes with central air conditioning, one of which was positive for all samples during both seasons. The remaining five positive Der p 1 samples were from four homes with evaporative coolers. For Der f 1, four of the positive samples originated in 2 homes with central air conditioning, and two of the positive samples originated in one home using an evaporative cooler. Two homes were positive for both Der p 1 and Der f 1, one with an evaporative cooler and one with central air conditioning.

Due to the low number of positive samples, Der p 1 and Der f 1 were combined for all subsequent analysis. Differences in mean allergen levels were not significant by season (F_1,207_ = 2.16, p = .1433), type of air conditioning (F_1,207_ = 2.05,p = .1533), occupant density (F_1,207_ = .27, p = .6059), nor average indoor humidity (F_1,207_ = .37, p = .5435). There was a significant relationship with age of home (F_1,207_ = 14.81, p = .0002), where higher allergen levels were associated with older homes. However, for all but one sample the allergens found in house dust were below the level considered clinically significant.

**Fig 1:**
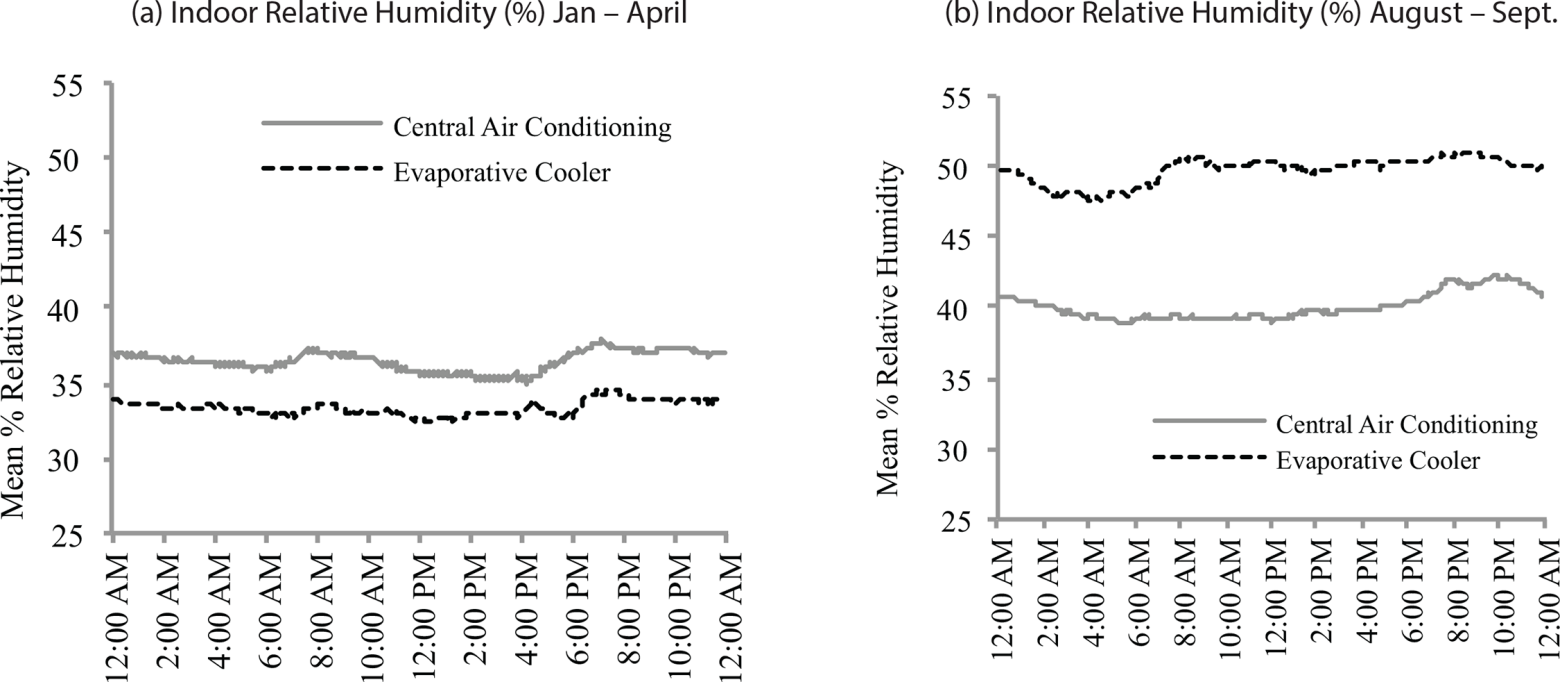
Average indoor relative humidity (%) by time of day for winter/spring and summer sampling periods. Average relative humidity was calculated by dividing the day into 288 5-min intervals, and taking the average of the readings for that time over the 72-hr sampling period.

Our analysis of mean indoor RH and temperature by season are reported in Table 3. There was not a significant difference in RH levels for central air homes between winter/spring and summer sampling (t_73_ = 1.62, p = 0.38), or between central air and evaporative cooler homes during winter/spring sampling (t_73_ = 1.21, p = 0.62). There was a significant difference between homes with evaporative coolers and homes with central air conditioning during summer (t_73_ = 4.00, p = 0.0008), and between homes with evaporative coolers from winter/spring to summer (t_73_ = 6.19, p < 0.0001). Indoor temperatures were significantly higher in both central air homes (t_73_ = 6.93, p < 0.0001) and evaporative cooler homes (t_73_ = 4.77, p < 0.0001) during summer compared to winter/spring sampling; however, there was no difference in indoor temperature between central air and evaporative cooler homes during winter/spring sampling (t_73_ = 0.62, p = 0.92), and summer sampling (t_73_ = 0.67, p = 0.91). Indoor RH trends by season and time of day are shown in Fig 1. For summer sampling, we observed a diurnal pattern where indoor RH increased during the daytime, and peaked during evening hours in both central air and evaporative cooler homes. There was a corresponding decrease in overnight RH in both types of homes. This pattern was more pronounced for evaporative cooler homes, where a larger morning peak was observed, possibly associated with daily evaporative cooler usage. For winter/spring sampling, both central air and evaporative cooler homes showed morning and evening RH peaks, with decreasing RH overnight.

**Table 3:** Indoor RH and temperature by season and type of air conditioning.

Type of Air Conditioning	Winter/Spring (Jan–Apr)	Summer	p-value^B^
		(Aug–Sept)	
	Mean (SE)^A^	Mean (SE)^A^	
Central Air			
RH (%)	36.41 (1.61)	40.10 (1.61)	0.38
Temp (°C)	20.16 (0.37)	23.78 (0.37)	< 0.0001
Evaporative Cooler			
RH (%)	33.34 (1.95)	49.73 (1.78)	< 0.0001
Temp (°C)	20.53 (0.45)	23.41 (0.41)	< 0.0001
p-value^B^			
RH	0.62	0.0008	
Temp	0.92	0.91	

^A^ Means and standard errors for RH and temperature are based on 72 hr samples collected with the monitor placed in a main living area of the home. Data were recorded every 5 min during winter/spring and summer sampling periods.

^B^ Comparisons based on t–tests with Tukey-Kramer adjustment.

**Fig 2:**
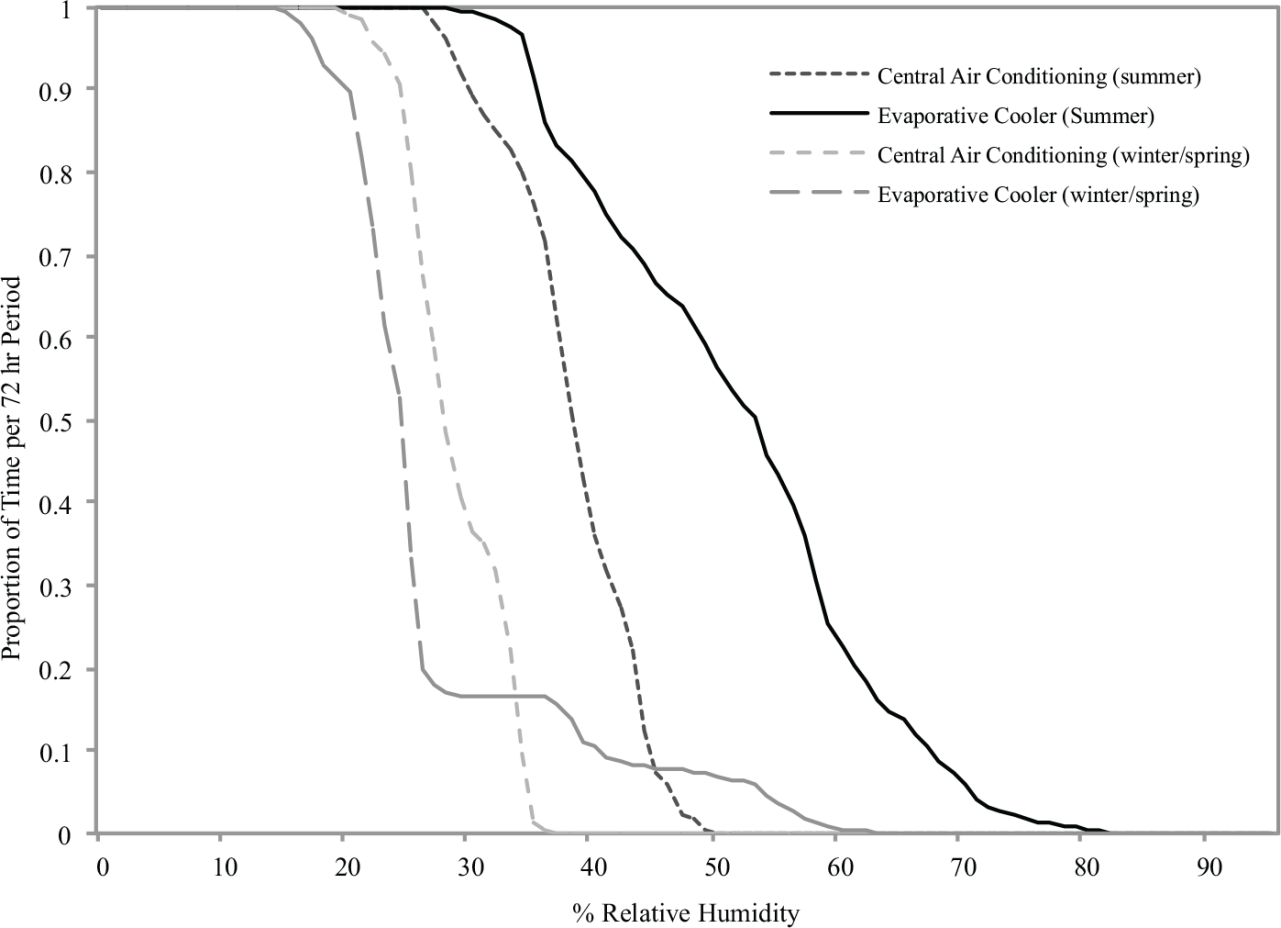
Proportion of time homes were at or above specified relative humidity levels by season and type of air conditioning. Relative humidity was measured in 5-min intervals over 72 hrs in each home. The proportion of time at or above a specific percent RH for each home was calculated by counting the number of observations at or above that level and dividing by the total number of observations for that home.

Considering that mites can survive and reproduce in conditions below the critical equilibrium humidity if provided with short-term moisture excursions of sufficient duration and intensity, an important measurement for mite growth in situ may be the number of minutes the home is at or above specific RH levels. In this study, we compared the number of minutes homes were at or above specific RH levels by season and type of air conditioning (Table 4). During the summer, evaporative cooler homes spent significantly more time at or above 55.0% and 65.0% RH per day compared to evaporative cooler homes in the winter/spring, and compared to central air homes during winter/spring and summer (p < 0.001). Trends in the proportion of time homes were at or above specific RH levels by season and type of air conditioning are shown in Fig 2. Evaporative cooler homes spent a greater proportion of time in the summer at higher RH levels compared to central air homes in the summer, and compared to all homes during winter/spring. Based on the low ambient RH found in this study, the proportion of time homes spent at or above 75.0% RH may be of particular interest. Evaporative coolers reached this level during the summer, but only for short durations, which may have been insufficient to rehydrate mites.

**Table 4:** Number of minutes/day homes were at or above specific RH levels.

Type of Air Conditioning	≥ 55%	≥ 65%	≥ 75%
	Mean (SE)	Mean (SE)	Mean (SE)
Central Air			
Winter/Spring	20.4 (53.8)	2.5 (17.4)	0.2 (2.9)
Summer	22.3 (53.8)	1.1 (17.4)	0.2 (2.9)
Evaporative Cooler			
Winter/Spring	47.3 (63.7)	2.6 (20. 6)	0.9 (3.5)
Summer	421.9 (58.5)^A^	77.1 (18.9)^A^	5.7 (3.2)^B^

^A^ Evaporative cooler homes spent significantly more time at or above 55 and 65% RH per day during summer than evaporative cooler winter or central air winter/spring or summer (p < 0.001).

^B^ Evaporative cooler homes spent a greater amount of time at or above 75% RH during summer than evaporative cooler winter or central air winter/spring or summer, however differences were not significant at the p = 0.05 level.

## Discussion

Understanding the relationship between housing factors and HDM growth is necessary to guide future environmental interventions aimed at limiting mite allergen exposures and associated diseases [1, 2]. Among various housing factors, air conditioning may have the greatest potential to modify the indoor environment to either limit or promote HDM growth conditions. The semi-arid climate found in Utah County, Utah provides an excellent environment to test the role of evaporative cooling on indoor RH and the presence of HDM allergens since the ambient RH is below the critical equilibrium humidity required for dust mite growth. Utah County is geographically located between the Colorado Front Range and Reno, NV where previous studies have shown an association between evaporative cooler use and elevated mite allergen levels and mite allergen sensitization in home occupants. A better understanding of the degree to which evaporative coolers influence RH trends in the home may help identify housing risk factors that contribute to HDM allergen exposure, as well as potential future intervention strategies.

With one exception, Der p 1 and Der f 1 levels found in this study were below the 2 μg/g of dust level considered to be clinically significant. The positive association between age of home and dust mite allergen levels may have been skewed due to one central air home that was 116 years old, and had the highest sample detected in this study (2.98 μg/g of dust for Der f 1). There was no significant association between allergen levels and type of air conditioning. This finding is likely explained by the limited increase in indoor RH produced by the evaporative coolers. Although there was a significant difference in indoor RH in evaporative cooler homes during the summer, levels were below the critical equilibrium humidity required for mite growth and reproduction. Furthermore, we tested the influence of short-term RH excursions, which have been shown in the lab to rehydrate mites even when mean RH is well below 50.0%. In this study, moisture excursions at or above 75.0% RH occurred for only a few minutes per day in evaporative cooler homes, whereas under laboratory conditions a minimum of 1.5–3.0 hrs per day was required to rehydrate female *D*. *pteronyssinus* mites sufficient to promote reproduction [22].

Our findings are in agreement with Tovey et al. (1997) who likewise reported low levels of Der p 1, and found no association between evaporative cooler use and allergen levels in homes in an arid region of Australia [28]. A plausible explanation for the low allergen levels reported here and by Tovey et al. is the presence of small numbers of non-introduced (indigenous) mites. A study of homes in the western U.S. intermountain states by Moyer et al. (1985) reported low numbers of indigenous mites (up to 40 mites/g of dust) in 25.0% of homes in Denver, CO, which has a climate similar to Utah County [14]. Nelson and Fernandez-Caldas (1995) similarly found mites in 17.2% of samples from homes in the intermountain states, half of which had fewer than 100 mites/g of dust [13]. Sparse populations of mites may survive in microenvironments within the home where they acquire moisture from occupants’ bodies for part of the day. Findings in this study support this theory. Low levels of Der p 1 and/or Der f 1allergens were found in 25.0% of study homes. We detected allergens most often in mattresses, upholstered furniture, and carpets adjacent to upholstered furniture. It is possible that small numbers of mites colonize furniture and mattresses where they receive sufficient moisture from occupants’ bodies to prevent desiccation.

Another possible explanation for the presence of low allergen levels is that mites were introduced into homes by furniture or mattresses brought in from locations with higher ambient humidity. Two central air homes and one evaporative cooler home had mattresses that were translocated from humid environments, including CA, OH, and NC. The mattress brought in from CA tested positive for both Der p 1 and Der f 1, and the mattresses brought in from OH and NC both tested positive for Der p 1. However, allergen levels in all three mattresses were below the 2 μg/g of dust threshold for sensitization. No homes with positive samples had upholstered furniture that had been translocated from humid regions. Thus, we believe the allergen levels detected in this study are predominantly from indigenous mite colonies.

Our results differ from previous studies that found a strong relationship between mite allergen levels and evaporative cooler use. Ellingson et al. (1995) found that 63.0% of Colorado homes with evaporative coolers tested positive for mite allergens during summer, with combined Der p 1 and Der f 1 mean levels of 11.02 μg/g of dust [18]. Vanlaar et al. (2001) reported mean Der p 1 concentrations of 11.29 μg/g of dust in homes with evaporative coolers in Australia [19]. In the Australia study, homes with evaporative coolers had a threefold higher concentration of Der p 1 than homes with central or no air conditioning. In contrast, mean Der p 1 and Der f 1 levels in our study were 0.11 and 0.37 μg/g of dust, respectively, and mean Der p 1 reported by Tovey et al. (1997) was 0.38 μg/g of dust from evaporative cooler homes [28]. All four studies evaluated the influence of evaporative coolers on mite allergen levels; however, the vastly different results may be due to environmental factors, such as RH, that were inconsistently measured, making direct comparison between the studies difficult.

Indoor RH is likely the most important measure needed to understand how evaporative coolers influence mite growth and subsequent allergen levels. However, this measure is not always reported [19, 28]. Ellingson et al. (1995) reported an average indoor RH of 59.1% during August in homes with evaporative coolers in Colorado [18]. This RH level is above the critical equilibrium humidity for HDMs, and may explain the high allergen levels reported in their study. However, indoor RH was measured using a participant-collect single-point sampling strategy in which home occupants recorded indoor RH once daily during evening hours. Single-point sampling can provide a rough estimate of mean indoor RH, but does not capture humidity trends in the home that may contribute to moisture excursions. In addition, RH trends measured by continuous monitoring in this and a previous study in Utah [21] show that indoor RH levels peak during evening hours. Thus, RH measurements reported by Ellingson et al. may have over-estimated the daily mean by a few percentage points. The conflicting findings among the few studies on this topic may be largely explained by temporal fluctuations in indoor RH related to evaporative cooler use. In order to make direct comparisons between studies, we recommend that future studies incorporate a continuous RH monitoring strategy that will give both mean humidity levels as well as the proportion of time spent at levels known to rehydrate desiccated mites.

Understanding the relationship between evaporative cooler use, indoor RH, and HDM allergen levels is also difficult due to limited information on home characteristics reported in previous studies. One explanation for the conflicting results may be found in the size of homes included in the studies. Homes sampled in this study were recruited from among university faculty and staff, and were relatively large and located in more affluent neighborhoods. Mean square footage of central air and evaporative cooler homes in our study were 3047 ft^2^ and 2277 ft^2^, respectively. It is possible that previous studies used significantly smaller homes with evaporative coolers than those used in this study, which may have contributed to higher RH levels. Smaller homes may be associated with higher occupant densities, which contribute to indoor RH. In this study we did not show that occupant density influenced Der p 1 and Der f 1 levels, but the relatively homogeneous sample of larger homes did not allow us to evaluate this influence across a spectrum of homes sizes. To facilitate better comparison of studies, data on home size and socio-economic status should also be reported in order to evaluate the influence these factors have on mite growth and allergen exposures.

This study was limited by the population of homes used for sample collection, which were relatively large and located in more affluent areas. To better understand the influence of evaporative coolers on indoor RH and mite allergen levels, it may be important to consider homes across a spectrum of sizes, socio-economic status, and other factors. In addition, summer sample collection extended into late September when outdoor temperatures tend to drop in Utah. If homes were sampled after occupants discontinued use of evaporative coolers, this may have artificially lowered the RH estimates reported in this study. However, in 2014, outdoor temperatures during September were unseasonable warm in Utah County until late in the month. For example, the last day of sampling occurred on September 25^th^. The maximum outdoor temperature recorded at the university weather station on this day was 32.7°C (90.8°F). Due to the unseasonably high temperatures, we assumed individuals continued to use air conditioning throughout the summer sampling period.

To our knowledge, our continuous monitoring strategy for RH is the most comprehensive attempt to understand the role of RH and dust mites in a semi-arid environment. One clear advantage to this monitoring strategy is that, in addition to providing mean RH, it provides data on humidity excursions in the home that may promote mite growth. Our work closely approximated the number of minutes that study homes were at or above specific RH levels, providing critical data in regards to optimal dust mite growth in a dry environment. One major limitation in understanding the role of evaporative coolers on HDM growth has been the lack of data on indoor RH. While our results did not show high allergen levels as found in similar studies, we can provide a plausible explanation as to why, based on mean indoor RH being below the critical equilibrium humidity, and insufficient intensity and duration of time for humidity excursions. Most importantly, RH excursions did not extend to 75.0% or above long enough to rehydrate desiccated mites, were they present in the home.

In conclusion, our data suggests that in a semi-arid climate like found in Utah County, low levels of Der p 1 and Der f 1 allergens may be present in approximately 25.0% of homes irrespective of whether evaporative coolers or central air conditioners are used, with Der p 1 being more prevalent. We believe these allergens originated from small numbers of indigenous mites that live in microclimates in the home where they find sufficient moisture to maintain water balance. Furthermore, we found that evaporative coolers significantly increased indoor RH during summer months, but levels were still below the critical equilibrium humidity required for mite growth. Humidity excursions approached levels that may rehydrate desiccated mites, but the duration of time was too short. Future research should consider smaller homes where evaporative coolers are used, and employ a continuous sampling strategy for RH.
